# Deep and Densely Connected Networks for Classification of Diabetic Retinopathy

**DOI:** 10.3390/diagnostics10010024

**Published:** 2020-01-02

**Authors:** Hamza Riaz, Jisu Park, Hojong Choi, Hyunchul Kim, Jungsuk Kim

**Affiliations:** 1 Department of Health Science and Technology, Gachon Advanced Institute for Health Sciences & Technology, Incheon 21999, Korea; hamza@bme.gachon.ac.kr (H.R.); jspark@bme.gachon.ac.kr (J.P.); 2 Department of Medical IT Convergence Engineering, Kumoh National Institute of Technology, 350-27, Gum-daero, Gumi 39253, Korea; 3 School of Information, University of California, 102 South Hall #4600, Berkeley, CA 94720, USA; 4 Department of Biomedical Engineering, Gachon University, 534-2, Hambakmoe-ro, Incheon 21936, Korea

**Keywords:** deep learning, densely connected networks, healthcare diagnosis, diabetic retinopathy, convolutional neural networks, fundus image analysis

## Abstract

Diabetes has recently emerged as a worldwide problem, and diabetic retinopathy is an abnormal state associated with the human retina. Due to the increase in daily screen-related activities of modern human beings, diabetic retinopathy is more prevalent among adults, leading to minor and major blindness. Doctors and clinicians are unable to perform early diagnoses due to the large number of patients. To solve this problem, this study introduces a classification model for retinal images that distinguishes between the various stages of diabetic retinopathy. This work involves deploying deep and densely connected networks for retinal image analysis with training from scratch. Dense connections between the convolutional layers of the network are an essential factor to enhance accuracy owing to the deeper supervision between layers. Another factor is the growth rate that further assists our model in learning more sophisticated feature maps regarding retinal images from every stage of the network. We compute the area under the curve, sensitivity, and specificity, particularly for messidor-2 and EyePACS. Compared to existing approaches, our method achieved better results, with an approximate rise rate of 0.01, 0.03, and 0.01, respectively. Therefore, computer-aided programs can help in diagnostic centers as automated detection systems.

## 1. Introduction

Diabetic cases, and specifically diabetic retinopathy cases, have been on the rise globally [1] leading to a major cause of blindness for young adults as well as older individuals (20–70 years) [2]. Even as a developed country, the U.S. has 93 million patients suffering from diabetic retinopathy (DR), and the number of such patients is increasing [2,3]. Diabetic retinopathy occurs as a result of retinal vascular diseases and abnormal blood flow in the retina. DR is usually characterized using four distinct levels for the disorder, including mild, moderate, severe, and proliferative diabetic retinopathy (PDR). Moreover, the mild, moderate, and severe DR categories are regarded as non-proliferative diabetic retinopathy (NPDR) whereas the neovascularization disorder is linked to PDR. The levels of DR are determined based on the difference in the ratio of diseases present, including microaneurysms, intraretinal hemorrhages (dot/blot), hard exudates, cotton wool spots, venous beading, and intraretinal microvascular abnormalities [2].

The motivation for various researches on medical images is purely related to the contribution to the diagnostic healthcare systems. Advances in the diagnosis for various diseases have been developed along with machine learning techniques based on medical images [4]. This is due to the requirement of a modern point-of-care (PoC) screening to promptly and exactly diagnose diseases outside the laboratory. Various applications installed in high-performance smart phones also have provided healthcare services to monitor the conditions of human body [5]. Such high-performance electronic devices and computer-aided programs make it possible for us to diagnose DR using deep learning techniques.

To prevent DR globally, various multipurpose diagnosis systems based on machine learning have already been proposed [6,7,8]. Initially, researchers used retinal fundus photographs for the detection of various eye diseases, using traditional machine learning methods including support vector machine (SVM), K-neighbor mapping, and random forest [9]. However, these algorithms are not general and lead to low accuracies as they require manual feature extraction. In contrast, deep convolutional neural networks (CNN) addressed the feature extraction problem; for large scale datasets, they also exhibited tremendous success in solving complex problems, including object detection, segmentation, and image translation [10]. CNNs also led to extraordinary achievements in the medical field, for the early diagnosis of diseases, tumors, and cancers in the human body [4].

In the past few years, numerous promising studies have been conducted on retinal image analysis. For this purpose, in [11], authors designed an algorithm to classify various retinal diseases with a relatively smaller database using a deep learning model called VGGNet. In [11], authors designed a system for types of diseases other than DR. Therefore, this technique allows a lot of room for error. Recently, Carson et al. used a deep learning model named GoogleNet for the detection of retinal lesions based on patches of input images [12]. In this work, each patch size was 128 × 128 × 3, which slowed it down tremendously in the training and inference process. Varadarajan et al. extracted completely novel information from retinal images using the ResNet architecture [13]. In [14], authors shared the performance of their automated detection system for DR and other eye diseases using deep learning. In addition, Gulshan et al. also used CNNs for the sole classification of DR for two different datasets producing state-of-the-art outcomes [15]. In [6], authors present a novel and cutting-edge system based on Inception V3. With this algorithm, researchers offer solutions for predicting various biometric factors from the retina including age, gender, body mass index (BMI), blood pressure, and smoker status. Generally, these articles faced common challenges in the analysis of retinal images. Indeed, a large number of uncertainty factors including blurriness, contrast, focus, distortion, whitening, and blankness [16] can create problems while training deep learning models, aiming at convergence.

### 1.1. Our Contribution

In this study, for the first time, we have used densely connected neural networks for the classification of DR. The motivation behind our approach was to deploy networks with more deep supervision to extract comprehensive feature maps from fundus images. In the literature, many research groups worldwide, including Google and Stanford, have already introduced VGGNet, GoogleNet, InceptionsNets, and ResNets, with fundus images for the classification of various diseases [6,9,11,13,14,15]. These articles indicate that every new deep learning model produced state-of-the-art outcomes in various domains of fundus images. Hence, for the first time, we introduce the idea of exploring deep and densely connected networks for the classification of DR, which further improves the performance of computer-assisted diagnosis for retinal images.

We propose a unique preprocessing step for the datasets for training and inference. Before the special preprocessing, we cleaned the messidor-2 and Kaggle datasets and similarly rearranged wrongly labeled data based on instructions of their websites [17,18]. Preprocessing also includes steps such as cropping window, data generation, and data normalization. We optimized densely connected neural networks (DenseNets) [19] to train on EyePACS images and test on messidor-2. We trained the models from scratch with an input resolution of 32 × 32 × 3. In the end, our idea improved the performance of the DR diagnosis system even for very-low-resolution images. Furthermore, we calculated the confusion matrix for class accuracy and performance.

### 1.2. Article Structure

The remaining article details are following: the preliminary steps, including data cleaning, rearranging, and data preprocessing, are discussed in Section 2. Data preprocessing involves three stages, namely the cropping window for efficient training, data generation to overcome class imbalance and overfitting, and data normalization. Section 3 illustrates the methodology in our approach to explore DenseNets with implementations. The results with confusion matrices, area under the curve (AUC), and a comparison table are reported in Section 4. A discussion on the results is presented in Section 5, and the conclusions of the study are presented in Section 6.

## 2. Data Preprocessing 

The analysis of the datasets [17,18] points to two major challenges. In messidor-2, there were many incorrectly labeled images, and, similarly, the Kaggle dataset is comparatively complex with numerous embedded camera artifacts. Therefore, we performed two steps to initiate data preprocessing.

### 2.1. Data Cleaning

**Figure 1:**
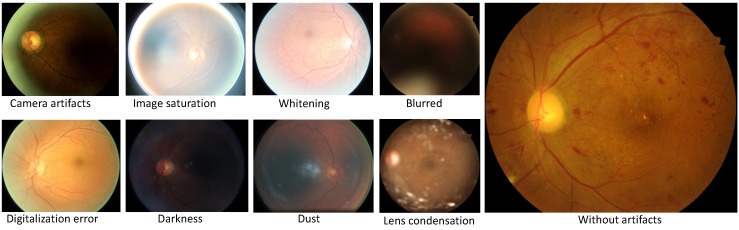
Various artifacts of the optical coherence tomography camera for the Eye Picture Archive Communication System (EyePACS) dataset. We cleaned the datasets from such defects, and these images are cropped from the original datasets.

Fortunately, messidor-2 is a cleaner dataset than Kaggle for DR. Before starting the training with EyePACS, it is important to scrub the data first. The dataset contains image artifacts, chromatic aberrations including image saturation, excess of whitening, blur and darkness, digitization error, dust, lens condensation, and without artifacts image. These categories of images are displayed in Figure 1. We manually separated these images from training and validation data. Authors in [6,16] also performed data scrubbing to produce novel outcomes but did not provide much details on it. This initial step is conducive to efficient training and inference on the target datasets.

#### Rearranging

The original messidor-2 has 1748 images, with several being labeled incorrectly [17], as indicated on its website. Prior to attempting inference with messidor-2, we should correct the labeling of these specific images. With the initial data scrubbing and rearranging, the dataset size becomes 1746, with five distinct classes. No mentions were found regarding mistakes in labeling in the EyePACS dataset for DR; because of this, only data scrubbing was performed there.

### 2.2. Preprocessing

In machine learning, amongst many essential components, preprocessing is key. Real-world datasets usually present themselves in raw form. Machine learning models perform poorly when datasets are not preprocessed as real-world data comes with various image resolutions, contrasts, illumination, orientations, etc. Similarly, the implementation of deep learning models on complex and large-scale datasets such as the ImageNet challenge [10], also involved special preprocessing from the perspective of the executed networks. In the same way, retinal images required preprocessing, because fundus images contain complex data with various fields of views (FOV), resolutions, illuminations, contrasts, smoothness, brightness, etc. Hence, in this paper, we also proposed a specific preprocessing methodology including image cropping, data generation, and normalization.

#### 2.2.1. Cropping Window

Fundus images are captured using optical coherence tomography (OCT) base cameras in the diagnosis centers. Traditionally, information on various anomalies of the retina is present in the center of the image with circular geometry [9]. Original OCT images carry an area of darkness, which indicates the presence of zero pixels around the circular retina. Previously, to achieve efficient training with deep learning models, various machine learning frameworks and libraries such as Keras, TensorFlow, Pytorch, and Darknet had supplied a generic function for image cropping. Our cropping is a window-based cropping, therefore, both datasets have different time during its procedure. For example, in the case of messidor-2, all dataset programs only took approximately 10 seconds.

Usually, the optimization of deep learning networks requires explicit image cropping to exclude redundant information from the input data. The efficient training of deep neural networks requires removing excessive zero-value pixels before the training process, for further convergence of the loss function and to avert the vanishing gradient issue [20]. Traditional cropping functions provide generic features for all types of datasets that can disturb the geometry of fundus images. In addition, cropping can cause a loss in the vital information regarding diseases because of which it can overlook prime features before the training process. Therefore, we propose a special cropping window for both datasets, which have images of various resolutions. We create windows of variable lengths dependent on the resolution of the images in the dataset. This type of cropping preserves crucial information on the datasets.

#### 2.2.2. Augmentation and Generation

Data augmentation is another essential step to yield well-trained deep learning models. As we know, most deep learning networks require a huge amount of data for sophisticated and generic results [10]. Data augmentation also protects deep learning networks from the most prevalent problem, namely overfitting [21]. Many techniques have already been developed in this vein, aside from augmentation [22]. Messidor-2 is a relatively tiny dataset in EyePACS and suffers from a class imbalance problem. EyePACS has a similar problem as well, before the training process, the total distribution of the size of the training set across classes is 25810, 2443, 5292, 873, 708 for normal, mild, moderate, severe, and PDR, respectively. Hence, to solve the class imbalance problem, we designed our custom augmenter with the functions of brightness, contrast, and rotation for the fundus images. The key point for this external data augmenter is that we applied these transformation functions to abnormal classes only. Our custom generator then created data in the specific directories with the label’s information.

#### 2.2.3. Data Normalization

Raw data are usually not presented in a standard shape to a deep learning algorithm. To improve accuracy and reduce error rates in the predictions of deep learning networks, several data normalization methods have been proposed [22]. This also minimizes the computational expenses of graphics processing units (GPUs) and central processing units (CPUs). Therefore, we also compute the means and standard deviation of the input datasets and the normalized whole fundus images with their standard deviation to lower the computational cost in the training process.

## 3. Methodology

**Figure 2:**
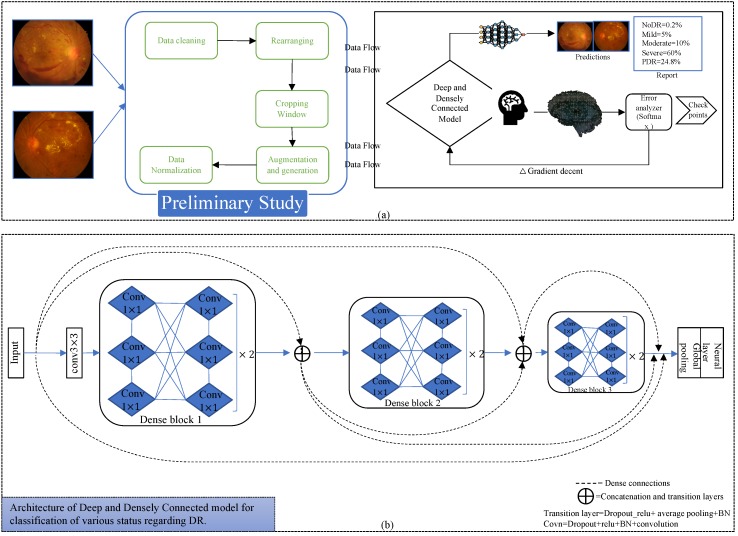
Process diagram for the execution of our method and details of the deep learning model. (**a**) Preliminary study including the initial manual data cleaning, rearranging dataset, designing the cropping window for various sizes of images to discard circular informative part, data augmentation for unbalanced classes to reduce overfitting, and data normalization. Moreover, the second part describes the general process of training and inference for datasets. (**b**) Architecture of deep and densely connected models for our implementation of a deep learning model to diagnose the status of diabetic retinopathy (DR). The model deploys three dense blocks with twelve convolutional layers in each and two transition layers with average pooling. It is a relatively deeper network to train; therefore, the dropout applies on each convolution layer to mitigate over-fitting with the model. The neural layer is a conventional dense (flatten) layer to convert feature maps to vectors that further contribute to the loss function. (The dotted lines mean concatenated connections between the layers of CNN, and the solid lines represents simple connection between the layers)

In this article, we exploited publicly available datasets including the messidor-2 and EyePACS, for the classification of DR status [17,18]. To achieve this goal, for the very first time, we evaluated a multiclass deep and densely connected model [19]; Figure 2 indicates the process diagram for such models. Similarly, to initiate training and inference, we followed the following key steps.

### 3.1. Data Preparation

This proposed deep learning network for the required conversion of original raw data of fundus images to useable form. Therefore, to run simulations using our approach, we first needed to follow preliminary steps including data cleaning, rearranging, implementation of cropping windows, augmentation and data generation, and data normalization. Following these steps, to initiate the training and validation processes for the deep and densely connected model, we split the data into respective sets. This constitutes the first block for starting an implementation procedure in the process diagram.

### 3.2. Deep and Densely Connected Networks

Deep and densely connected networks [19] are the successors of ResNets [23], but they contain a distinctive connectivity in the deeper layers referred to as dense connections. Figure 2 also represents the dense connectivity patterns between the layers of the CNN. In [19], authors explained the many advantages of such networks over ResNets [23] and InceptionNets [24], especially for deeper supervision, reduced complexity, parametric efficiency, lower computational power requirements, and higher accuracy.

Deep and densely connected networks [19] comprise various dense blocks, and each block contains combinations of many 1 × 1 and 3 × 3 convolutional layers (conv) with dense connectivity. Our model consists of three dense blocks, each having twelve convolutional layers. A transition block is another essential element in the implementation of such networks; it includes batch normalization [25], relu activation [26], 1 × 1 conv, 2 × 2 average pooling layer, and concatenation layers. A transition layer is implemented after every dense block; in addition, before the first dense block, we also implemented a 3 × 3 conv layer as in the original article. After the last dense block, we implemented a global average pooling, fully connected or conventional neural network layer which also known as the dense layer, and a softmax layer as a loss function.

#### Selection of Hyperparameters

The optimization of deep learning models requires an efficient tuning of hyperparameters. A model depends on these parameters, and these can be different across models. The common hyperparameters for DenseNets are the growth-rate (K), dropout, depth of network, number of dense blocks, learning rate, learning decay ratio, weight decay, compression factor, bottleneck layer activation, optimizer, momentum, epochs, batch size, and weight regularization. To optimize our network for the training over fundus images, we used K = 12, depth of network = 40, batch size = 64, epoch = 350, and three dense blocks. We set the learning rate = 1 × 10^−3^, learning decay ratio = 0.1, weight decay = 1 × 10^−4^, and L2 regularization on weights. The compression factor and bottleneck layer activation are also implemented in our designed network. To optimize the weights of the model, we implemented a stochastic gradient descent optimizer having a momentum of 0.9 with Nesterov mode. We kept the dropout at 0.2, to reduce overfitting [27].

### 3.3. Implementation

The execution of dense blocks with various hyperparameters allows us to move further in the implementation process. The details are as follows:

#### 3.3.1. Training and Inference

The training is performed on the EyePACS dataset after the preliminary steps. We trained the deep and densely connected networks on 71,913 balanced fundus images and validated on 17,979 images. Training continued on an Nvidia GPU GTX 1080ti with described setting of parameters. During training, the first conv layer extracts initial feature maps which are further manipulated in the first dense block to extract low-level features with a 32 × 32 × 168 resolution. Only two transition layers with average pooling are employed after the first and second dense block. The first transition layer performs the down sampling for low-level features to 16 × 16 × 168. After every conv layer the feature maps grow with a factor of twelve within the dense block and concatenation helps combine all of them. Likewise, the second dense block extracts mid-level 16 × 16 × 312 features maps, and the final transition layer, with the help of average pooling, down samples them to 8 × 8 × 312. The final dense block generates high-level features for the fundus images 8 × 8 × 456 feature maps. In the end, the global average pooling and neural layer (dense) are applied and the softmax loss function helps to learn representative predictions for fundus images. The training and validation losses are 0.163 and 0.265, respectively, for fundus images to classify five levels of DR.

We evaluated the performance of the dense block-based model on the messidor-2 dataset with 1747 images and the EyePACS dataset with 17,978 images.

#### 3.3.2. Threefold Cross Validation, Checkpoints, and Graphs

In machine learning, trained models can be overfitted for deeper networks. Therefore, we applied threefold cross validation at the time of training [28]. In the architecture of the network, we applied a dropout factor for every conv layer with a 0.2 dropout rate [27]. The threefold validation and dropout assist the relatively deeper networks and thereby prevent them from overfitting. Moreover, the purpose of checkpoints is to save the best weights at the time of training with respect to loss and accuracy curves. During cross validation, our algorithm generates checkpoints after every five epochs on the basis of model loss and accuracy. Furthermore, the saved weights facilitate the inference process on new datasets. In the end, the proposed approach calculates the graphs of the loss function, accuracy, and performance of the model on new datasets by storing history of the models and tensor-board.

### 3.4. Calculation of Performance

In this study, we were able to calculate the confusion matrix for each class at the time of inference. Moreover, our algorithm extracts sufficient information from the testing data, such as area under the curve with average and as well as per class AUC. The next section provides the results in the form of confusion matrix and AUC.

## 4. Results

**Figure 3:**
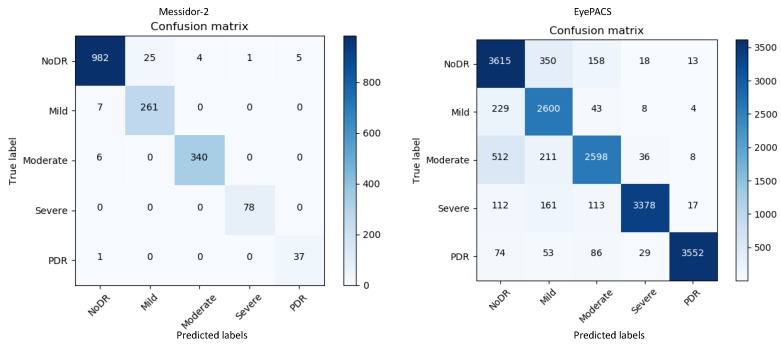
Results of the confusion matrices for both datasets, using a trained deep and densely connected model. Actual and predicted labels are displayed on the y-axis and x-axis, respectively.

The model evaluates the outcome on messidor-2 and EyePACS images in the form of a confusion matrix; furthermore, it evaluates the performance table including precision, sensitivity, specificity, F_1_ score that means 2×(precision×recall)/(precision+recall), and AUC. Figure 3 represents the confusion matrices for both datasets. The confusion matrices in Figure 3 show that for messidor-2, the model yields adequate results compared with those for EyePACS as it is a tiny and clean dataset. By contrast, EyePACS is sharply noisy, unclean, and contains a large number of images; therefore, the model underperforms slightly there. Moreover, the proposed method can generate a report table that delivers important information regarding each class in the retinal datasets to support comprehensive analysis. Table 1 describes the performance of the proposed model on the messidor-2 datasets; it uses various mathematical equations to evaluate the report on the input data. 

Table 1 and Figure 3 provide key information on the retinal images for messidor-2 specifically; mild-DR indicates that there is a less than 5% chance to contract the DR disease, hence a normal image and a mild-DR are shared almost similar features, a promising potential reason behind the relatively lower precision.

Applying the same method, a report for the EyePACS data is reported in Table 2. Precision, sensitivity, and specificity are the key metrics for checking the accuracy of a model. For our method, we evaluate the F1-score, which checks the accuracy of the test data in the form of harmonic average specifically for imbalance datasets.

In this study, we assessed the performance metrics with a five-class dataset; therefore, to describe the mathematical equations for each parameter, let us consider a generic 5 × 5 matrix, as given below:

$$\mathrm{Predicted}\;\mathrm{Results}\;\mathrm{Actual}\;\mathrm{Results}\;\left[\begin{array}{cccccc} Class & 1 & 2 & 3 & 4 & 5\\ 1 & A_{1,1} & A_{1,2} & A_{1,3} & A_{1,4} & A_{1,5}\\ 2 & A_{2,1} & A_{2,2} & A_{2,3} & A_{2,4} & A_{2,5}\\ 3 & A_{3,1} & A_{3,2} & A_{3,3} & A_{3,4} & A_{3,4}\\ 4 & A_{4,1} & A_{4,2} & A_{4,3} & A_{4,4} & A_{4,5}\\ 5 & A_{5,1} & A_{5,2} & A_{5,3} & A_{5,4} & A_{5,5} \end{array}\right]$$

It represents any confusion matrix with five classes from one to five. The x-axis and y-axis represent the predicted and actual class information, respectively. It first performs calculations for overall accuracy in Equation (1). The ratio of correct predictions over the aggregate number of data points is known as the overall accuracy, and the overall accuracies for messidor-2 and EyePACS are 0.97 and 0.88, respectively. Similarly, precision is defined as the ratio of true positive (T_P_) values for a specific class with the combination of true positive and false positive (F_P_) values from the confusion matrix. F_P_ is the total sum of all elements in a column related to a specific class.

(1) $$Overall\;Accuracy=\frac{Correct\;Predicted\;values}{Sum\;of\;total\;values}=\frac{Sum\;of\;diagonal\;elements}{Sum\;of\;total\;values}\;=\frac{A_{1,1}+A_{2,2}+A_{3,3}+A_{4,4}+A_{5,5}}{Sum\;of\;total\;values}$$

The final equation for precision can be written as in Equations (2) and (3):

(2) $$Precision=\frac{True\;positive\;\left(T_{P}\right)}{True\;positive\;\left(T_{P}\right)+False\;positive\;\left(F_{P}\right)},$$

(3) $$Precision\;for\;class\;3=\frac{A_{3,3}}{A_{3,3}+A_{1,3}+A_{2,3}+A_{4,3}+A_{5,3}}.$$

From the matrix, to find the precision specially for class 3, T_P_ is A_3,3_ and F_P_ is the combination of all the elements in the third column of matrix, specifically A_1,3_, A_2,3_, A_4,3_, and A_5,3_, excluding T_P_. After applying this formula, the algorithm evaluates the precision for all classes and computes the average values of 0.95 and 0.88 for messidor-2 and EyePACS, respectively. Furthermore, sensitivity is another essential metric that conveys the accuracy of a machine learning model. It is the ratio of T_P_ and sum of T_P_ with false negatives (F_N_). In contrast with F_P_, it is the sum of all the elements in the row of the target class excluding T_P_. Equations (4) and (5) represent the sensitivity, also known as true positive rate (TPR).

(4) $$\begin{array}{ll} Sensitivity & =true\;positive\;rate\;\left(TPR\right)\\ & =\frac{True\;positive\;\left(T_{P}\right)}{True\;positive\;\left(T_{P}\right)+False\;negative\;\left(F_{N}\right)} \end{array}$$

(5) $$Sensitivity\;for\;class\;3=\frac{A_{3,3}}{A_{3,3}+A_{3,1}+A_{3,2}+A_{3,4}+A_{3,5}}$$

Equation (4) is a generic formula to determine the sensitivity or *TPR* (Recall) and Equation (5) describes the sensitivity for class three only. Similarly, after calculating the sensitivity for messidor-2 and EyePACS, the average values are found to be 0.98 and 0.94, respectively. Specificity is another metric representing the accuracy of a machine learning model, and it is calculated by dividing the true negative (T_N_) by the combination of T_N_ and false positive (F_P_), as in Equation (6). T_N_ is sum of all the elements except the row and column of the specific class. Similarly, let us consider a scenario where we attempt to find the specificity for class 3; $T_{N_{3}}$ is the true negative rate for class 3, it is shown in Equation (7) and FP is the same as Equations (3) and (4). It is also known as the true negative rate (TNR). The average specificities for messidor-2 and EyePACS are 0.98 and 0.97, respectively. Furthermore, precision and sensitivity help evaluate the F1 score which is another crucial metric for expressing the accuracy of unbalanced datasets. This is expressed in Equation (9), as follows:

(6) $$\begin{array}{ll} Specificity & =true\;negative\;rate\;\left(TNR\right)\\ & =\frac{True\;negative\;\left(T_{N}\right)}{True\;negative\;\left(T_{N}\right)+False\;positive\;\left(F_{P}\right)}, \end{array}$$

(7) $$True\;negative\;for\;class\;3=T_{N_{3}}=A_{1,1}+A_{1,2}+A_{1,4}+A_{1,5}+A_{2,1}+A_{2,2}+A_{2,4}$$

(8) $$Specificity\;for\;class\;3=\frac{T_{N_{3}}}{T_{N_{3}}+A_{1,3}+A_{2,3}+A_{4,3}+A_{5,3}},$$

(9) $$F_{1}score=\frac{2\times Precision\times Recall}{Precision+Recall}$$

The average values of the F1 score for messidor-2 and EyePACS are 0.97 and 0.88, respectively. The results indicate that the model provides relatively low results for [18]; this could be attributed to the fact that the first three classes share almost the same features.

The equations above and various representations to express the results are all equally important. However, at the final stage, the model calculates the area under the curve (AUC) for each class and its average as well. The rationale for checking the AUC for every individual class is linked to the analysis of the impact of the trained model on each class. AUC is computed using a receiver operating characteristic (ROC) curve, which is a graph conveying the performance of a classification model. The ROC curve plots involve two metrics such as *TPR* and false positive rate (*FPR*). We already have TPR, but *FPR* is computed using Equation (10), as follows:

(10) FPR=1−TNR

**Figure 4:**
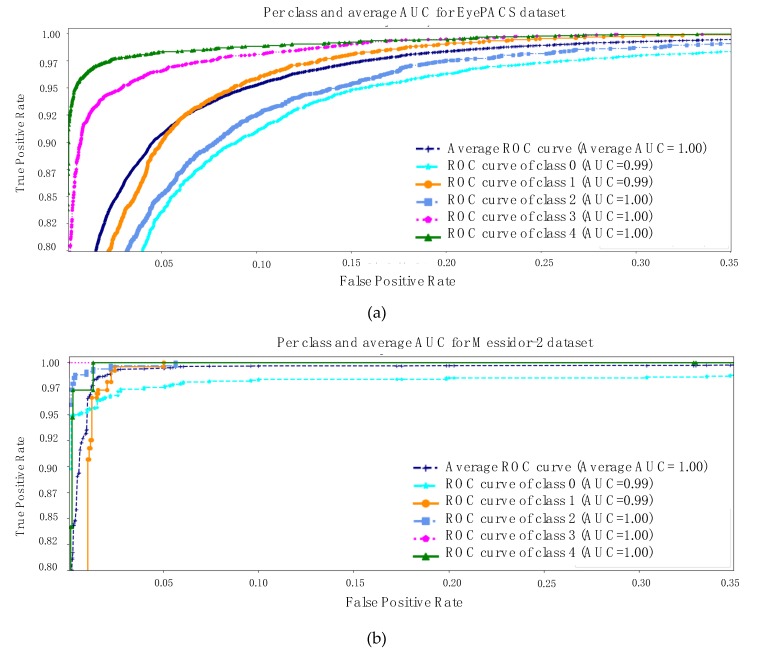
(**a**) AUC results for EyePACS dataset, (**b**) AUC result for Messidor-2. The receiver operating characteristic (ROC) curve for each individual class was determined to compute the averages.

Finally, AUC stands for area under the ROC curve. Therefore, Figure 4 is describing the AUC for both datasets [17,18]. The same key points are highlighted, specifically messidor-2 is a simpler and smaller retinal dataset than EyePACS and the first three classes of each dataset are sharing a few similar features. With all the evidence, the model exhibits an average AUC of 1 and 0.98 for both datasets separately.

## 5. Discussion

**Figure 5:**
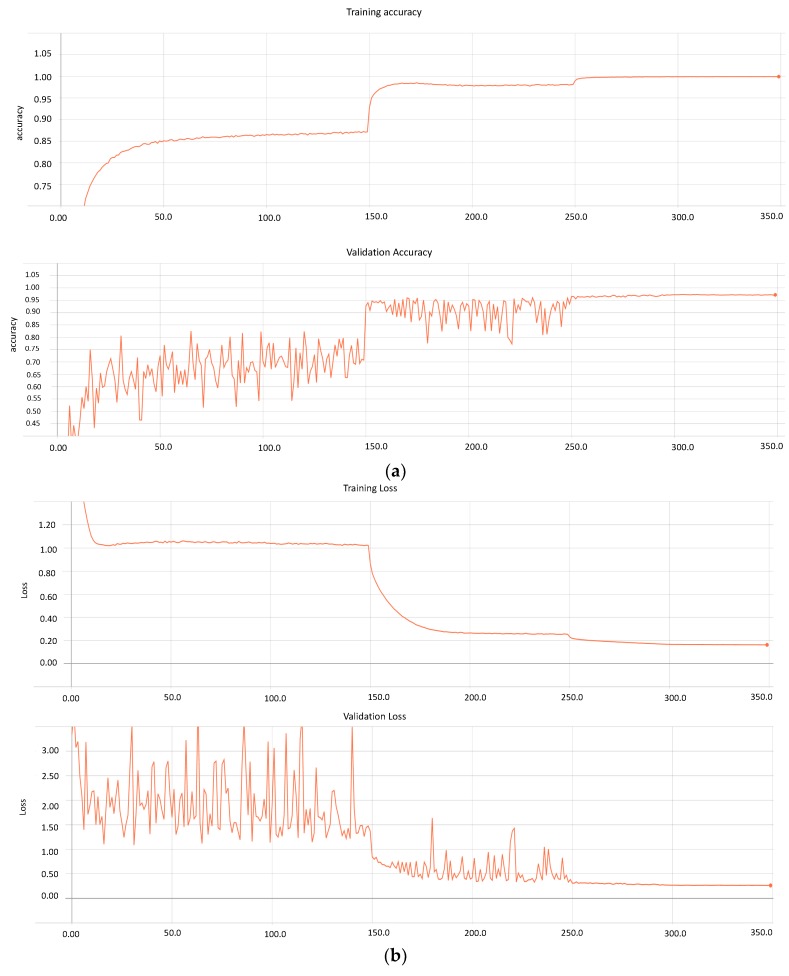
(**a**) Represents the accuracy of model for training and validation. Our deep and densely connected model runs for 350 epochs. (**b**) Represents the loss of model for training and validation process. It shows how loss of model reduces across epochs.

In this study, we investigated the classification of the cases of DR disease, using deeper and dense networks. This method can perform diagnosis based on the various status of the DR images. To reach this goal, we trained deep and densely connected networks with distinct sets of hyper parameters, as discussed in Section 3 (Methodology). During the training process, the method determines the graphs for the model’s loss and accuracy for 350 epochs. Figure 5a,b presents both metrics for the trained model.

Similarly, the model performs training on 71,913 images and validation on 17,979. Furthermore, based on the graphs, it can be seen that after 300 epochs, the model loss and accuracy remain constant, which can result in overfitting. Therefore, our approach stores various weights at distinct learning rates with a loss of 0.163 and 0.265 for the training and validating data, respectively. Conversely, the accuracy of the model on messidor-2 and EyePACS is 0.99 and 0.97, respectively.

### Comparison with Other Related Approaches

We compared our method with state-of-the art approaches [15,29,30,31] for messidor-2 and EyePACS images. Table 3 depicts the prospective comparison. Previously, researchers have performed experiments by using various frameworks, including GoogleNet and InceptionNet v3. However, in our study, we adopted a different network and achieved state-of-the-art results. For messidor-2, the model provides the highest results as compared to [12,29,31], specifically for AUC, sensitivity, and specificity. The model exhibits a significant improvement in sensitivity, with a score of 0.98. Similarly, the deep and densely connected model also delivers comparative enhancement in sensitivity for the EyePACS dataset with respect to [15,29]. Furthermore, the AUC and specificity are similar to that of other methods reported in literature. From Table 3, it can be seen that one of the main characteristics of our model is the higher stability as compared to other approaches. For messidor-2, [15] delivers similar outcomes, particularly for AUC and specificity. However, in the case of sensitivity, it exhibits significantly lower values. Similarly, for EyePACS, the results of [15] appear broadly similar to those of the proposed model, except for sensitivity where our model achieves 0.94 vs. 0.90 in [15]. Furthermore, for [29], the sensitivity is almost identical but the other two parameters are lower than those of our method.

Furthermore, special cropping is only a preprocessing method to reduce the loss and increase the accuracy. In virtue of the combination of preprocessing and deep learning model, we can increase overall accuracy for both datasets up to 0.97 and 0.88, respectively which we mentioned in the results section.

Another driver for the enhanced accuracy is linked to the relatively deeper and complex nature of networks such as ResNets [23], compared with others in literature [24,32]. Usually, such models require more memory and parameters for training; however, in our case, the model is deeper than others but still with low requirements, unlike ResNet [19]. However, even after accounting for these strengths, our model is still a deeper model, which means that to perform training and inference more hardware resources such as GPUs are needed. Therefore, to increase accuracy, our model provides deep supervision with a trade off in system requirements as nowadays, faster and more powerful GPUs, such as the RTX family, are available. This experiment was conducted on a GTX 1080ti which is six times slower than the RTX 2080ti [33].

## 6. Conclusions and Future Research

This paper introduced a state-of-the-art deep learning model for the classification of DR from images. In this work, we also proved that deep and densely connected networks have the potential to yield deeper supervision, which further secures the more relevant feature maps alone. Based on this, the accuracy improves across a wide range; for instance, in messidor-2, our model provides the highest AUC, sensitivity, and specificity. Another key aspect of our approach is the data scrubbing and the special cropping windows; both help the model to learn quickly with discriminant features regarding each abnormality. Moreover, this method also supplies deep and comprehensive analysis for each class of images by providing a confusion matrix and generating reports with various representations of essential parameters. Finally, it indicates that our method improves accuracy and reduces the loss; the performance gains are clearly visible from the comparison table as well. As a result, we can assert that we pioneered the use of deep and densely connected models for the diagnosis of DR by analyzing retinal images.

In the future, we plan to examine various datasets for the detection of lesions to highlight damaged pixels in the images, which can be flagged as a segmentation problem instead of a classification one. Furthermore, we have already assembled a fully connected deep and densely connected network, which will be a new tool leading to a single segmentation model for all types of lesion detection. Although the current work is related to the classification of DR, in the near future, this research will be helpful to design a general deep learning model that can exactly detect defective areas or abnormal pixels on the retinal images. In order to reduce the complexity, traditional works have used specific datasets that are targeted at similar types of diseases. In this work, we have proposed to make a common dataset that can segment various diseases with only one model. This method could be useful for early state PoC screening services in the future.

## Figures and Tables

**Table 1:** Illustrates a report on messidor-2 with precision, sensitivity, specificity, F_1_ score, and area under the curve (AUC). The last column shows the images available for the individual classes in the dataset.

Class	Precision	Recall (Sensitivity)	F_1_ Score	AUC	Specificity	Images Per Class
No-DR	0.99	0.97	0.98	0.99	0.98	1017
Mild-DR	0.91	0.97	0.94	0.99	0.98	268
Moderate-DR	0.98	0.98	0.99	1.00	0.99	346
Sever-DR	0.99	1.00	0.99	1.00	0.99	78
PDR	0.88	0.97	0.93	1.00	0.99	38
Average-score	0.95	0.98	0.97	1.00	0.98	Total = 1747

**Table 2:** Represents the performance of the model on the EyePACS dataset.

Class	Precision	Recall (Sensitivity)	F_1_-Score	AUC	Specificity	Images Per Class
No-DR	0.80	0.87	0.83	0.97	0.93	4154
Mild-DR	0.77	0.90	0.83	0.98	0.95	2884
Moderate-DR	0.87	0.77	0.82	0.97	0.97	3365
Sever-DR	0.97	0.89	0.93	0.99	0.99	3781
PDR	0.99	0.94	0.96	1.00	0.99	3794
Average-score	0.88	0.94	0.88	0.98	0.97	Total = 17,978

**Table 3:** Express a clear comparison to other classification-based methods with deep and densely connected network.

Methods	AUC	Sensitivity	Specificity	Datatype
Gulshan et al. [15]	0.99	0.87	0.98	Messidor-2
Sayres et al. [29]		0.91	0.947	Messidor-2
Carson et al. [30]		0.95		Messidor-2
This proposed method	1.00	0.98	0.986	Messidor-2
Gulshan et al. [15]	0.99	0.90	0.98	EyePACS
Sayres et al. [29]	0.84	0.945	0.90	EyePACS
This proposed method	0.982	0.94	0.97	EyePACS
